# Sports Coaches’ Knowledge and Beliefs About the Provision, Reception, and Evaluation of Verbal Feedback

**DOI:** 10.3389/fpsyg.2020.571552

**Published:** 2020-09-15

**Authors:** Robert J. Mason, Damian Farrow, John A. C. Hattie

**Affiliations:** ^1^Melbourne Graduate School of Education, The University of Melbourne, Parkville, VIC, Australia; ^2^Institute for Health and Sport, Victoria University, Melbourne, VIC, Australia

**Keywords:** feedback, feedback reception, coaching, coaching effectiveness, instruction, pedagogy, sport coaching

## Abstract

Coach observation studies conducted since the 1970s have sought to determine the quantity and quality of verbal feedback provided by coaches to their athletes. Relatively few studies, however, have sought to determine the knowledge and beliefs of coaches that underpin this provision of feedback. The purpose of the current study was to identify the beliefs and knowledge that elite team sport coaches hold about providing, receiving and evaluating feedback in their training and competition environments. Semi-structured interviews conducted with 8 coaches were inductively analyzed, revealing three broad themes: thinking and learning about feedback, providing feedback, and evaluating feedback. Findings revealed a detailed array of knowledge about feedback across a wide range of sub-topics. Coaches saw feedback as a tool to improve performance, build athlete confidence, help athletes to monitor progress, and as a tool to improve their own performance. Novel insights about evaluating an athlete’s reception of feedback, and tailoring feedback for individual athletes, were provided by coaches. The findings also highlight areas in which future coach education offerings can better support coaches to provide effective feedback.

## Introduction

Coaches are thought to require strong procedural knowledge about the pedagogical strategies required to help athletes learn effectively (Nash and Collins, 2006) in addition to possessing specific knowledge about their sport. Recent studies have investigated the knowledge of coaches regarding sport-specific topics such as resistance training (Harden et al., 2019), swimming technique (Morris et al., 2019), and talent identification (Roberts et al., 2019). However, a major gap in this growing body of literature about coach knowledge concerns the use of pedagogical techniques such as feedback in coach practice. Therefore, the research question for consideration in this paper concerns what coaches know and believe about the provision, reception, and evaluation of coach-athlete verbal feedback. It is acknowledged that feedback in an elite sporting setting is not limited to that provided verbally by a coach. Although important in the overall context of learning design in an elite sporting setting, the role of the coach as a practice designer and facilitator of athletes seeking their own feedback (Woods et al., 2020) is not the primary focus of the current paper. A large body of coach observation literature consistently finds that verbal feedback represents one of the most common coach behaviors observed (Partington and Cushion, 2013), with rates of over 60 feedback messages per game reported recently in an elite setting (Mason et al., 2020a). As such, an investigation into verbal feedback appears important to determine the knowledge that coaches hold about this major element of their practice.

Feedback is widely regarded as a frequently used and high-impact strategy to progress a learner from current to goal performance (Kluger and DeNisi, 1996; Hattie, 2009). Many studies have quantified and analyzed coach feedback in both training and performance settings (e.g., Partington et al., 2015; Halperin et al., 2016). However, an area receiving less attention is the investigation of coach knowledge and beliefs underpinning the feedback they provide (Smith and Cushion, 2006). Supplementing the large body of empirical evidence on coach feedback in practice with an investigation of the experiential knowledge of expert coaches is considered to be an important direction for improving pedagogical practice (Greenwood et al., 2014). Qualitative investigations of coach practice such as interviews (Tinning, 1982; Potrac et al., 2002) may assist in providing greater detail about the contexts and constraints that coaches operate under in reality (Kahan, 1999). Recent studies examining coach knowledge (e.g., Roberts et al., 2019) have not yet filled the gap in the area of pedagogical strategies such as feedback.

In education, a large body of evidence exists on teacher beliefs and links to their subsequent practice. Reviews of this literature suggest that teachers hold a variety of beliefs about their pedagogical practice that vary across context and cultures, and that these beliefs are usually related to the pedagogical practices they adopt (Fang, 1996; OECD, 2009). Importantly, it is suggested that efforts to improve teacher practice must take into account the perceptions and beliefs of teachers (Putnam and Borko, 1997). Similarly, an understanding of coach beliefs about feedback is an important step in ultimately improving coach feedback practices, and coach education in general (Côté et al., 1995). There is evidence that myths from the field of education have been adopted by sports coaches; 62% of surveyed coaches in the United Kingdom believed that individuals learn better when they receive information in their preferred learning style (Bailey et al., 2018). This is contrasted with many major reviews (e.g., Pashler et al., 2009) which concluded that there is “no adequate evidence base” (p. 105) for the effectiveness of learning styles. More broadly, several authors have lamented the absence of a belief in evidence-based approaches to pedagogy in high performance coaching (Rushall, 2003; Davids et al., 2017).

Current evidence on coach knowledge about feedback suggests a wide spectrum of philosophies, ranging from the highly coach-controlled to a more facilitative and athlete-centered approach (Côté et al., 1995; Potrac et al., 2002; Smith and Cushion, 2006). In the former category is a case study of an expert English soccer coach, who reported beliefs toward providing feedback such as “they’ve got to be told what is expected of them” (Potrac et al., 2002, p. 191). The coach expressed a desire to be in control of his players during training because his job security ultimately depended on game-day success, and this was reflected in the feedback he provided. In another study, gymnastics coaches reported preferring to provide their athletes with feedback constantly (Côté et al., 1995), reflecting that it was important that their athletes “know where they are regularly” (p. 82). These high levels of coach control are contrasted with evidence that some coaches adopt a highly athlete-centered approach to feedback. Smith and Cushion (2006) found that expert English soccer coaches used silence strategically during in-game coaching to allow players to make decisions without an overly prescriptive approach. Coaches also reported not wanting to overload athletes with information, preferring to provide a small number of simple prompts. Allowing the athletes to experiment without coach intervention, and asking an athlete to self-evaluate before providing feedback, were strategies mentioned by the more athlete-centered coaches interviewed in the Côté et al. (1995) study. Across studies, a similar spectrum is seen when coaches are asked to consider feedback valence; some coaches report using up to 90% negative feedback (Côté et al., 1995), while others reported a more balanced approach (Smith and Cushion, 2006). This evidence suggests large variations in coach beliefs about effective feedback practices, which may reflect the unique challenges (Lyle, 2002) represented by the different contexts in which coaches work. For example, and of interest to the current study, is the differences in feedback between team and individual sport coaches. It appears that determining the gap between current coach practice and “best practice” for feedback as reported in the literature is an important task in improving the feedback that coaches give.

A potential challenge for this area of research is evidence to suggest that coaches can be inaccurate when reflecting on their use of feedback. For example, rowing coaches were observed providing verbal feedback to their athletes during training, and then an hour later they were asked about the feedback they provided (Millar et al., 2011). It was found that coaches overestimated desirable feedback patterns by between 5 and 40%; coaches tended to underestimate their use of highly prescriptive instruction, and overestimate their use of questioning to allow athletes to evaluate performance or describe affective feeling about performance. Coaches also appear to over-report the provision of tactical information over technical information compared to actual rates observed in the feedback they provided to athletes (Pereira et al., 2009; Millar et al., 2011). Additionally, coaches and athletes show low agreement when asked to recall the types of feedback provided by the coach, with the highest correlation in one study *r* = 0.26 between athlete and coach perceptions (Smoll and Smith, 1980). These findings highlight the importance of triangulating coach interview data with observational data where possible.

An area not commonly considered in feedback research is the reception of feedback (Anderson, 2010); much time and effort has been spent on determining the quality and quantity of feedback provided, without considering its reception and subsequent action by a receiver. Little is known about the ways in which coaches evaluate their feedback to determine its reception and use by their athletes. Barriers to the successful reception of feedback by athletes include discrepancies in interpretations of feedback between the provider and the receiver (Liberman et al., 2005; Adcroft, 2011). Other barriers include variations in the characteristics of the feedback receiver such as working memory capacity (Buszard et al., 2017), or the receiver’s self-efficacy to receive and act on feedback (Narciss and Huth, 2004). There have been numerous calls in the literature (Langley, 1997; Potrac et al., 2000) for research designs to consider the athlete’s ability to receive feedback, but relatively few studies have done so (for an example, see Mason et al., 2020b). Despite the importance of considering individual athlete factors, there is evidence to suggest that coaches may have high confidence but low accuracy when judging their athletes’ mental skills (Leslie-Toogood and Martin, 2003).

### Present Study

The literature on coach beliefs and knowledge about verbal feedback is still in its infancy. The variation observed in what coaches know and believe regarding the provision of feedback may be caused by factors such as experience, context, or perceived job pressure. Additionally, a major gap in the area is an understanding of how coaches consider athlete factors such as the capacity and disposition to receive verbal feedback from a coach. Supplementing the large body of empirical evidence on coach feedback in practice with an investigation of the experiential knowledge of expert coaches is considered to be an important direction for improving pedagogical practice (Greenwood et al., 2014).

The purpose of this study was to qualitatively determine the knowledge and beliefs currently held by elite sport coaches with regard to the provision, reception, and evaluation of verbal feedback in training and competition environments. Given the proposition that coaches must possess a strong understanding of the pedagogical strategies required to help athletes learn effectively (Nash and Collins, 2006), along with evidence that coaches may hold some misconceptions about pedagogy (Bailey et al., 2018), it was hypothesized that there would be much variance in the beliefs and knowledge about feedback, with varying degrees of support from academic evidence.

## Materials and Methods

### Participants

Eight coaches currently employed in a high performance team sport setting were recruited for the study. Recruitment was limited to coaches who had at least 5 years of experience coaching in a high performance setting. For the purpose of the study, this was defined as a professional national-level league or international representative (i.e., national team) setting. This definition is broadly consistent with similar previous studies that have sought to investigate high performance coaches (Rynne and Mallett, 2012; Morris et al., 2019). The sampling procedure was aligned with a purposeful sampling approach (Creswell, 2013), to ensure that expert coaches who have experience with a high-performance team sport environment could provide insight into the research questions. Coaches were aged between 32 and 52 years (*M* = 42.63, SD = 6.55), and had a mean experience level of 9.75 years (SD = 3.20) in a high performance setting. Coaches represented the sports of Australian rules football (2), rugby (2), basketball (2), soccer (1) and field hockey (1). Five coaches were involved in elite national-level competitions with senior athletes, two were involved with elite youth national representative teams (under 18 age group), and one was involved with a senior national representative team. Six of the coaches had participated as athletes in the sport they coached to a high performance level, while two had not. Demographic information about the participating coaches is provided in Table 1.

**TABLE 1:** Coach demographic information.

Coach	Gender of coach	Sport	Gender of athletes	Level
Coach 1	M	Basketball	M	Senior
Coach 2	F	Basketball	F	Elite youth
Coach 3	M	Australian football	M	Senior
Coach 4	M	Australian football	M	Senior
Coach 5	M	Soccer	F	Senior
Coach 6	M	Field hockey	M/F	Elite youth
Coach 7	M	Rugby	M	Senior
Coach 8	M	Rugby	M	Senior

Participants were recruited via email or phone. At the time of the interview, participants were provided with a plain language statement and consent form, and were given the chance to answer questions about the study before enrolling. All participants were assured of anonymity and informed that participation was entirely voluntary. Ethics approval was obtained from the Melbourne Graduate School of Education’s Human Ethics Advisory Group (Ethics ID: 1851890.1).

### Interview Guide

To assist with consistency between interviews, a semi-structured interview guide was constructed. General information sought from the participant at the beginning of the interview included current role, time in current role, total years of experience coaching in a high performance setting, and any relevant experience as an athlete. These questions served to provide important demographic information, and were also used as rapport-building opening conversations to introduce a relaxed, conversational style to the interview (Côté et al., 1995). Researchers are encouraged “to keep uppermost in one’s mind the fact that the interview is a social, interpersonal encounter, not merely a data collection exercise” (Cohen et al., 2011, p. 421), so care was taken to develop this rapport initially.

Questions from the main part of the interview focussed specifically on the research question; a list of questions can be found in Table 2. Consistent with a semi-structured interview approach, probes (e.g., “Are there any other ways you know the feedback has been received?”) were used when participants provided relevant but incomplete information, to seek a deeper response, or to ensure the clarity of the response. Any new topics that emerged during the course of the interview were explored by the interviewer, consistent with methods adopted in similar semi-structured interview research in sport (Potrac et al., 2002).

**TABLE 2:** Interview questions.

1	How important is providing feedback to your players in your role?
2	How often do you give feedback to your players? In what settings?
3	For what purpose do you typically use feedback?
4	How much of your feedback is positive vs. negative/constructive?
5	Do you tailor your feedback based on the individual athlete? If so, how?
6	Do some players respond better to feedback than others? If so, how?
7	How do you know when an athlete has received the feedback?
8	Do you think it’s best to provide feedback to the athlete or let them solve performance problems by themselves?
9	Do you give good feedback? If not, what prevents you from giving better feedback?

### Procedure

The study protocol was explained to participants, who were then offered an opportunity to ask questions about their involvement in the study and assured of the confidentiality of their identity and responses. Informed consent was then obtained from the participant via a hard-copy form. All interviews were recorded on an Apple iPhone 6S, and an Apple MacBook Pro internal microphone was used as a second backup recorder. All interviews were conducted by the first author, who has undertaken undergraduate and postgraduate training in qualitative and quantitative research methodology. Interviews were conducted primarily in person (*n* = 5), with a further 3 interviews conducted via phone or Skype; research suggests that Skype and phone interviews can be an appropriate replacement for in-person interviews where geography is a limiting factor (Deakin and Wakefield, 2014). Interview duration was between 14 and 46 min (*M* = 27.25, SD = 10.42). Within 24 h of the interview, the interviewer transcribed the interviews verbatim into a Microsoft Word document. All participants were provided with a copy of the interview transcript within 1 week of the interview, and asked to check the transcript for accuracy and clarity.

### Data Analysis

Transcripts were uploaded into NVivo for analysis. Given the precedent in coaching literature for an inductive approach to qualitative interview data (Potrac et al., 2002; Rynne and Mallett, 2012), data analysis in the current study also adopted an inductive approach. The process of inductively coding data followed the methods outlined by Côté et al. (1993). First, interview transcripts were read and assigned a label to begin the general process of categorizing the data. At this stage, the primary focus of coding was to organize rather than to interpret. Following a first round of coding, all labels were compared and assigned a broader category, a process known as *creating categories* (Côté et al., 1993). For example, any text tagged with “positive feedback” or “descriptive feedback” was assigned to a category called “types of feedback.” In completing a similar procedure, Rynne and Mallett (2012) acknowledged that categories remained flexible due to the need for adjustment as coding took place; in the current study, many instances of re-coding took place as themes emerged and developed. The final step in the analysis process involved the naming of final themes, along with the generation of a narrative to accompany each theme in the context of the research question for presentation in this article (Braun and Clarke, 2006). Categories discussed by at least half of coaches (i.e., 4 or more), or that were considered theoretically important for the research area, were included in the final themes.

## Results

The three higher-order categories that emerged throughout the inductive analysis procedures were *thinking and learning about feedback, providing feedback*, and *evaluating feedback*. The major sub-themes of each category are presented in Table 3 below. The following section will detail the major findings within each category and sub-theme with respect to the range of knowledge and beliefs held by the high performance coaches interviewed. Where relevant, quotes from interviewees are included with the pseudonyms Coach 1 through to Coach 8. The gender-neutral pronoun “they” has been used throughout to conceal the gender of the coach.

**TABLE 3:** Emerging themes and sub-categories following the process of inductive analysis.

*Thinking and learning about feedback*	*Providing feedback*	*Evaluating feedback*
Roles for feedback (8) – To improve performance (6) – To build confidence (5) – To monitor progress (2) – To help coaches improve (2) Learning about giving feedback (5) – Mentors (2) – Peer learning (3) – Shortcomings of formal coach ed. (2)	Feedback valence (7) Feedback quantity (8) Allowing athletes to problem-solve (5) Structures/frameworks (3) – Goal setting (2) – Individual Performance Plan (1) – “Shit sandwich” (1) Timing of feedback (3) Barriers to giving better feedback (8)	Methods for evaluating feedback reception (8) – Observing change in performance (7) – Questioning (4) – Statistics (2) Factors influencing feedback reception (8) – Personality variables (4) – Overloading the athlete (5) – Amount of feedback (6) – Terminology (2)

*Numbers in parentheses represent the number of coaches who contributed ideas to each theme/sub-category.*

### Thinking and Learning About Feedback

One of the major categories identified through the collation of sub-themes was the way in which coaches conceptualize, learn about, and reflect on their use of feedback. Sub-themes under this category include: coach beliefs about the roles of feedback, and sources of learning about providing feedback.

#### Roles of Feedback

Coaches held varying beliefs about the role and purpose of feedback in their coaching practice that fell into four main themes: improving performance, monitoring progress, helping coaches to improve, and building athlete confidence. A strongly held belief was that coaches see feedback as a major tool for improving individual and team performance. Coach 7 reflected on the importance of feedback for improvement, stating that “if you don’t get feedback, you don’t really know how you’re tracking and how you’re developing.” Coach 7 went on to clarify that they saw feedback as a tool to help both athletes and coaches grow, suggesting that feedback is conceptualized not only as something to be given by coaches, but also received and used to improve coaching practice.

Aside from the role of feedback as a means for improving performance, 5 interviewees also discussed the importance of feedback for building confidence and providing reassurance when both individual and team performance was not ideal. Coach 4 spoke of the importance of showing positive video feedback to their team following a loss in order to re-motivate the group. This was also discussed by Coach 1, who said that they would often ask video analysts in their organization to just cut up some positive footage because a player’s “confidence is so bad right now.” The role of feedback as reassurance also extended to a competition setting, with Coach 2 reflecting that “I think 50% of my job on game day is to tell them [the athletes] that everything is okay, and that they’re going okay.” Coach 3 took a different approach to the motivational role of feedback, sharing that they often provided overly positive feedback to one athlete with the hope that it may induce competitiveness and prompt other athletes to “strive for similar feedback.”

#### Learning About Giving Feedback

Coach 5 believed that having a mentor was an effective method for improving their use of feedback, stating that “the best thing that any new coach could do is partner up.” A common theme was that coaches trusted advice from experienced peers, with Coach 6 explaining that “I’d probably like to go from experience and what’s worked for them [another coach] rather than going for something completely drastic and new.” Coach 8 reflected critically on formal coach education courses, stating that “I enjoy doing them, just the piece of paper doesn’t do much for me,” while also speaking of the importance of informal learning for their improvement as a coach. It appears that coaches already working at the high performance level see limited benefit in obtaining formal accreditation, instead valuing the informal learning opportunities presented by collaborating with peers or mentors.

### Providing Feedback

A second major category emerging from the interview data relates to beliefs and knowledge about the practicalities involved with providing feedback. In this section, sub-themes include: feedback valence, feedback quantity, providing feedback vs. allowing athletes to problem-solve, structures and frameworks, timing, and barriers to giving better feedback.

#### Feedback Valence

One of the most commonly discussed beliefs amongst the interviewed coaches was the ideal ratio of positive to negative (also referred to by the coaches as “constructive,” “growth” and “room for improvement”) feedback. There was a common acknowledgment from interviewed coaches that rates of positive to negative feedback vary according to the coach’s personal style and the context in which they operate. Coach 1 recalled an experience of working under a head coach who was “a little more old school” and “doesn’t think much about being more positive… if he has something to say about it [a video clip], he’s going to say it,” while also acknowledging their own style to be more “modern” and responsive to the needs of the athlete. Half of the coaches articulated the belief that providing too much negative feedback was detrimental to athlete performance. For example, Coach 8 reported striving to show athletes positive examples to guide them toward desired behavior, rather than negative examples that show an athlete performing poorly. Coach 3 believed that mostly positive feedback should be used during competition, with “constructive” feedback left for breaks in competition or during training. Like many other coaches, Coach 3 believed that the motivational benefits of positive feedback could enhance performance during competition, with negative feedback believed to cause doubt or impact the concentration of the athlete.

#### Feedback Quantity and “Overloading” Athletes

All coaches spoke about feedback quantity, with over half discussing their struggle find the right balance between providing enough feedback to ensure the most salient points were covered, but also keeping feedback quantity within a range that was manageable for athletes to use. Coach 7 used an analogy to describe their approach to deciding on feedback quantity: “If I tried to throw you 10 tennis balls, you’d probably catch 2–3… If I throw you 2–3 tennis balls, you’ll probably catch 2–3… Players can only retain a certain amount of information, and chunking up that information from smaller bits is really important.” The philosophy of Coach 2 was similar: “2–3 [pieces of feedback] tops.” Coach 4 reported taking an individualized approach to deciding on the amount of feedback provided to athletes, considering motivation to be an important factor in determining how much feedback athletes prefer: “[My approach is] if you want the info I’ll give it to you, but I’m not going to chase you either. If I’m chasing them they’re probably not going to look at it anyway. They’ve got to drive it and want it themselves.”

#### Providing Feedback vs. Allowing Athletes to Problem-Solve

The influence of training design frameworks such as the constraints-led approach, where coaches are encouraged to design environments in which athletes are able to solve problems rather than simply being told by a coach (Renshaw et al., 2016), was clearly seen in coach responses. This was summarized by Coach 8, who observed the following:

They’re the ones out there on the field in the heat of the battle. For me to come in and tell them everything… well, I’m not out there to solve their problems on game day, on the field. I just want to steer them and guide them to come up with the answers.

Coach 5 was stronger in their phrasing, believing that “you’re not winning the game from the coaches’ box.” Coach 3 took this philosophy into their training design, reporting that they often manipulated the amount of feedback provided during a training session to encourage athletes to problem-solve without coach intervention: “I’ll say to the coaching staff, we’re not holding their hands through any of the session, don’t say anything to them… they have to find their own way.” When reflecting on their work with less experienced coaches, Coach 2 believed that a novice coach is more likely to adopt an “I tell” coaching mentality, in which coaches will “tell them [the athletes] what they see without giving the student/player an opportunity to think of their own answers.” Coach 4 believed that this led to negative outcomes for both coach and athlete, whereby the coach “can easily get frustrated when they give advice and then they don’t see that change in behavior from the player.”

#### Structures and Frameworks

Three of the eight coaches discussed a more formal approach to providing feedback, detailing the frameworks they have in place for providing regular feedback to their athletes. This was most common in coaches who worked with a national squad, where athletes typically train in their local environments when not with the national team. For example, Coach 6 reported providing regular feedback in the context of an individual performance plan (IPP), in which 3–4 goals are set in consultation between the athlete and coach before a tournament begins. Coach 6 then works with athletes during the course of a tournament to provide feedback against the goals outlined in the IPP. After tournament completion, Coach 6 triangulates feedback from themselves as head coach, their assistant coaches, and self-assessment from the athlete themselves before generating a new IPP for their local context.

Other coaches reported less formal structures for providing feedback. Coach 7 reported their use of the colloquial “shit sandwich” method of providing feedback: “Start with a positive, then a negative, then finish with a positive. I was taught that way back when. In some ways when I do my game reviews, I structure it a bit like that. Here’s some things we did really well, here are some things we need to fix up, look at efforts where we did really well off the ball. I still haven’t gone too far away from that.”

#### Timing of Feedback

Three coaches explicitly mentioned the importance of timing feedback for maximum impact on their athletes. Coach 3 believed that feedback was often most effective if provided before an opportunity to implement it in performance, choosing to provide feedback directly before training when practical, in order to see immediate change in performance. Coach 3 suggested that:

Feedback at the end of the session is good, just general feedback or how they performed or whatever, but if there is a particular thing that you need them to try and get, I have found it’s gotta be right before the next session so it’s fresh.

Coach 8 relayed similar sentiments, believing that feedback “on-the-run” during training or competition was more easily implemented than feedback given in a video review setting away from the performance environment.

#### Perceived Barriers to Giving Better Feedback

All coaches reflected on challenges they faced in their day-to-day roles that may not be conducive to providing feedback that is in line with their views of “best practice.” One of the most commonly reported barriers was time. Coaches 3 and 6 both worked with national representative squads, where intensive tournament play at international level is often interspersed with months away from athletes while they train and play with their local teams. Coach 3 reflected that “you might only see them [athletes] for a few days at a pre-tournament camp, and then it’s another 2 months until another camp.” Coach 6 spoke of the importance of checking in on individual athletes in their local environments, to ensure continuity and consistency of feedback across the course of a year. Coach 8 mentioned the difficulty associated with having up to 15 players under their care during a season, admitting that some players don’t sit down with a coach to review footage and receive feedback for “a couple of weeks.” To circumvent this, the coach provides feedback in a group setting more regularly.

### Evaluating Feedback

A major focus of this paper is on determining the beliefs, knowledge and reported approaches taken by coaches with regard to feedback reception. Coach 6, a former school teacher, was a particularly strong advocate for more closely considering the reception of feedback by athletes:

I think athletes, or kids in school, they almost need to be trained or given methods of what is feedback and how to receive feedback. We think about how we deliver it a lot, and we put a lot of effort into ourselves – hopefully – in that area, but it’s actually a skill to receive feedback.

The following section presents coach reflections on: methods for evaluating feedback reception, and factors influencing the reception of feedback by an athlete.

#### Methods for Evaluating Feedback Reception

Coaches were varied in the extent of their responses to questions relating to the reception of feedback by athletes, and typically fell into one of two groups: one group of coaches appeared to prefer a practical approach to evaluating feedback reception by way of observing physical performance, while another group reported using pedagogical tools such as questioning for assessing player knowledge and retention of feedback.

The most common response from coaches was that performance in competition is a reliable measure of the effectiveness of feedback; for example, Coach 1 reflected that “the way you know if it’s been 100% effective is if they do what you told them, at the end of the day, on the court.” However, two coaches also believed that this method of evaluating feedback was not completely reliable, citing extraneous variables such as skill errors or athletes choosing not to buy in to the coach’s strategic changes as possible reasons that observing performance may not accurately reflect the reception of feedback.

Another commonly reported method for seeking evidence that feedback has been received by athletes is through questioning or otherwise designing a learning environment where athletes can provide verbal evidence of understanding to the coach. Coach 5 explained their approach to providing video feedback, stating that they believed the feedback had been received “… if they can take you through a different piece of vision or a different scenario from the one where we first might have unearthed an issue or whatever it was, and they can talk it back to you.” Coach 8 believed that an athlete-centered approach to video feedback meetings was needed in order to evaluate feedback reception:

If I’m doing all the talking, I don’t know if they’re understanding what I’m saying. I ask a lot of questions, or I put up a clip and get them to tell me what they’re thinking. That way we can sort of find somewhere in between where we can meet.

Other reported methods for evaluating feedback reception include reading non-verbal markers such as body language, and analyzing in-game statistics.

#### Athlete Characteristics Influencing Feedback Reception

Coaches were asked to report any characteristics of their athletes that are perceived to act as facilitators or barriers to the athletes receiving feedback. Four coaches described attitude or entitlement problems observed in their athletes. This reportedly led to a reluctance to receive and accept feedback, particularly negative or constructive feedback. Coaches suggested that ego and previous experience with overly positive feedback were the main contributors. For example, Coach 2 shared their experience with athletes who are “overwhelmed by positive feedback from people around them, and they believe the hype.” Coach 3 reflected that the most difficult athletes to coach are:

… the ones that have coaches back home that have told them what they’ve wanted to hear all of the time. They haven’t had a coach who has been constructive with them, and they haven’t got family that say “you still need to work on this.” They have surrounded themselves with “yes” people.

Other coaches spoke of the “participation trophy era” (Coach 5), alluding to a phenomenon whereby junior athletes receive trophies for simply entering an event, not just for winning. It appears that a major challenge for coaches is adjusting the approach they take when providing feedback to athletes who exhibit a reluctance to receive feedback.

Another belief frequently mentioned by coaches in this area related to knowing the athlete as an individual and acknowledging the ways in which they prefer to receive feedback. Coaches alluded to this being the “art” of coaching; for example, Coach 8 reflected, “that’s coaching, isn’t it? Knowing who wants what.” The importance of differentiating feedback for individuals was acknowledged by Coach 7, who ran pre-season surveys with all their athletes to determine their preferences for receiving feedback.

The most commonly reported methods of differentiating feedback for players involved tailoring the amount or the valence of the feedback. Coach 1 reported that their assistant coaches were mindful of the amount of video feedback that athletes preferred. For example, “[this athlete] doesn’t like watching film, so let’s just keep it short, 3–4 clips.” Coach 6 believed that certain athletes had a natural feel for the game and didn’t benefit as greatly as others from video feedback: “To some guys the footage can just become a drag for them. Some of those natural players, you start showing them all that and putting them into a box – well that’s not what they’re good at.” Coach 6 believed that giving these types of athletes feedback in a training environment may be more productive than in a video review session. Similarly, coaches believed that certain athletes benefited more from either positive or negative feedback, differentiating based on preference. Coach 3 spoke of their experience working with athletes who varied in their need for feedback: “[player], you just had to tell her how great she was all the time… others, you could be a lot harder on.” Coach 4 believed that most of their athletes preferred hearing positive feedback, but observed that some athletes in their squad have “an ability to have a bit more of a ‘dressing down’ type of feedback.”

## Discussion

The aim of the study was to determine the knowledge and beliefs currently held by high performance sport coaches with regard to the provision, reception, and evaluation of feedback. The findings presented above illustrate the multifaceted and complex nature of current coach knowledge about feedback. The notion that coaches must possess knowledge of pedagogical strategies required to help athletes learn effectively (Nash and Collins, 2006) was supported by a rich array of information collected about the many strategies that coaches use for providing and evaluating feedback in their roles. As predicted, there were also some areas in which coach knowledge about feedback did not align with current evidence.

A number of ideas emerging from the interview data align with prior research. The most fundamental of these was the belief that feedback is a useful tool for improving both individual and team performance. Coaches considered feedback to be a vital part of their role and a commonly used pedagogical tool, implemented with the intention to improve player performance. Links between feedback and performance are reflected throughout a range of feedback literature (Kluger and DeNisi, 1996; Hattie and Timperley, 2007; Randell et al., 2011). Importantly, the notion of receiving feedback as a coach in order to improve coaching practice was also mentioned, reflecting Hattie and Clarke’s (2018) emphasis on feedback being a two-way process between receiving and giving. The idea of using student assessments as feedback on teaching is not new in education (Nicol and MacFarlane-Dick, 2006), but the current study also shows that coaches seek feedback from athletes to evaluate their impact in much the same way.

Coaches articulated preferences for informal learning sources when asked about how they might upskill themselves in the area of feedback, with five coaches referring to learning from peers or a more experienced coach as a preferred way to seek improvement. One coach reflected critically on formal learning sources such as accredited coach education courses. These sentiments align with evidence from previous studies on coach education, where typical findings are that informal learning sources such as discussions with peers are preferred over formal courses (Erickson et al., 2008; Stoszkowski and Collins, 2016). One reason for this preference, with particular relevance to the interview data, is that formal coach education courses often do not allow for substantial participant interaction (Demers et al., 2006). Striking a balance between allowing for the sharing of experiences between coaches, while also advocating for evidence-based feedback practices that do not promote neuromyths (Bailey et al., 2018) or folk pedagogy (Bruner, 1996), appears an important endeavor for future coach education offerings. Working with a mentor (McQuade et al., 2015) or coach developer (North, 2010) may be an avenue for further exploration, given the learning preferences articulated by coaches in the current study.

An area yielding novel data in the current study relates to the use of questioning by coaches to check for feedback reception. Previous studies suggest that coaches ask few questions (Potrac et al., 2002), and that coaches tend to overestimate their use of questioning when asked to self-report (Millar et al., 2011). Coaches in previous studies also report not wanting to ask too many questions due to a desire to avoid appearing indecisive or lacking expertise (Potrac et al., 2002). Despite this, evidence suggests that questioning paired with feedback can have a positive effect on performance (Chambers and Vickers, 2006). A commonly reported method for evaluating feedback reception in the current study was through questioning, with five coaches suggesting that they check for player understanding of feedback through using open-ended questions. Coaches also reported creating athlete-centered learning environments in which athletes were encouraged to show evidence of their understanding through analyzing video with coach feedback withheld. Athlete-centered coaching has been noted in the literature as an effective method for improving performance and motivation of athletes (Light and Harvey, 2017). An important avenue for future research appears to be matching self-reports of teaching behaviors with observations to verify their accuracy. However, the data collected in the current study provides evidence of commonly held knowledge that there are a number of ways to check for feedback reception.

An emerging topic in sport psychology research relates to entitlement attitudes displayed by some athletes (Dorsch and Etheredge, 2017). This theme presented clearly in the coach interviews, particularly when coaches were asked to discuss barriers experienced when providing negative feedback to athletes. Four coaches discussed the “participation trophy era,” referred to by others as “the selfie age” (Gilbert, 2016), as a potential influence on the reluctance of a certain generation of elite athletes to receive negative feedback. One potential recommendation from this finding is that coaches may need more support in overcoming particular athlete personality characteristics when providing negative feedback. Managing egos and expectations about the nature of feedback (particularly with respect to valence) appears important for ensuring that athletes are willing to receive feedback. Prominent theories on attribution (e.g., Dweck, 2000; Boekaerts, 2006) may provide some value in assisting coaches to provide feedback that is less likely to be interpreted as a threat to perceived competence, and more likely to be seen as an opportunity for growth by the athlete. This may be an avenue for future coach education offerings.

One area in which reported coach knowledge was at odds with evidence concerns the strategies or frameworks that coaches use to deliver feedback, particularly with regard to feedback valence. While coaches generally gave their views on an ideal ratio of positive to negative feedback, some coaches mentioned the notion of a feedback sandwich or “shit sandwich,” in which a piece of negative feedback is “sandwiched” between two pieces of positive feedback. Although it is claimed the feedback sandwich technique can have affective benefits such as building rapport with the feedback receiver (Dohrenwend, 2002), evidence suggests that the feedback sandwich does not impact post-feedback performance (Parkes et al., 2013) in a sample of medical students, and can encourage the feedback receiver to overlook negative feedback and reach artificially positive conclusions (Shute, 2008). While the generalization of these findings into the sporting context should be made with caution, it provides a viable avenue for future research.

### Limitations

The evidence presented in the current study provides an insight in to the current knowledge and beliefs of high performance coaches about verbal feedback. However, the use of coaches working at the highest level limits the generalisability of findings to coaches working at other levels. To determine the ways in which knowledge about feedback develops over time, a comparison between novice and expert coaches may be beneficial. Widening the scope to investigate differences between individual and team sport coaches would also provide additional information about how coaches use feedback in different contexts. An acknowledged limitation of the sample used in the current study is the small number of coaches recruited overall, and the brevity of some of the interviews. This represents a major challenge associated with working alongside high performance coaches while in-season. Several barriers with recruitment and retainment of participants were experienced throughout the data collection phase of the study, which may be alleviated in future work by collecting data early in pre-season when competition is not intense. Crucially, it should be recognized that verbal feedback is just one source of feedback available to athletes; future studies could consider the interaction between verbal and other sources of feedback. Finally, future research should further investigate the relationship between what coaches say they do in interview studies, and what they actually do while coaching. This is especially important given evidence that coaches can be inaccurate when reflecting on the feedback they provide (Pereira et al., 2009; Millar et al., 2011).

### Practical Applications

The findings of this study provide information about what expert high performance coaches know and believe about feedback. As such, this information may be useful as a model to coaches working at other levels, as it represents the current “best practices” that are adopted by these expert coaches. Below are some potential practical applications arising from this study:

• Coaches should consider that feedback has various roles: to improve performance, to monitor progress, to help coaches improve, and to build athlete confidence.

• Coaches prefer to learn about feedback from peers and mentors; this should be reflected when designing future coach development opportunities.
• Coaches should consider feedback quantity, and try to avoid “overloading” athletes with many feedback messages.
• Coaches should consider ways of evaluating the reception of feedback by their athletes. These include observing performance changes, and pedagogical tools such as questioning or allowing athletes to teach or explain a concept.
• Coaches should consider various athlete characteristics that may help or hinder feedback reception. These may include athlete attitudes and preferences.

## Conclusion

This study provides insight into the knowledge and beliefs of high performance coaches with regard to the provision, reception, and evaluation of feedback in training and competition environments. It adds important qualitative detail to the myriad of observational studies of coaches providing feedback, filling a gap commonly identified in previous research. The findings suggest that coaches possess a highly nuanced understanding of the ways in which the power of feedback can be harnessed in their individual contexts and, importantly, evaluated for reception and effectiveness. The findings also highlight areas in which future coach education offerings can better support coaches to provide effective feedback.

## Data Availability Statement

The raw data supporting the conclusions of this article will be made available by the authors, without undue reservation.

## Ethics Statement

The studies involving human participants were reviewed and approved by Human Ethics Advisory Group, Melbourne Graduate School of Education, University of Melbourne. The patients/participants provided their written informed consent to participate in this study.

## Author Contributions

RM conceptualized the study, undertook the data collection, data analysis, and wrote the manuscript. DF provided feedback on the study conceptualisation, data analysis, and manuscript. JH provided feedback on the study conceptualisation, data analysis, and manuscript. All authors contributed to the article and approved the submitted version.

## Conflict of Interest

The authors declare that the research was conducted in the absence of any commercial or financial relationships that could be construed as a potential conflict of interest.

## References

[B1] Adcroft A. (2011). The mythology of feedback. *Higher Educ. Res. Dev.* 30 405–419. 10.1080/07294360.2010.526096

[B2] Anderson R. (2010). “Augmented feedback - the triptych conundrum,” in *28th International Conference on Biomechanics in Sports 2010. Marquette: ISBS Conference Proceedings Archive*, eds Jensen R. Ebben W. Petushek E. Richter C. Roemer K. (Marquette, MI).

[B3] Bailey R. P. Madigan D. J. Cope E. Nicholls A. R. (2018). The prevalence of pseudoscientific ideas and neuromyths among sports coaches. *Front. Psychol.* 9:641. 10.3389/fpsyg.2018.00641 29770115 PMC5941987

[B4] Boekaerts M. (2006). “Self-regulation and effort investment,” in *Handbook of child psychology: Vol. 4. Child psychology in practice*, 6th Edn, eds Renninger K. A. Sigel I. E. (Hoboken, NJ: John Wiley), 345–377.

[B5] Braun V. Clarke V. (2006). Using thematic analysis in psychology. *Q. Res. Psychol.* 3 77–101. 10.1191/1478088706qp063oa 32100154

[B6] Bruner J. S. (1996). *The Culture of Education.* Cambridge, MA: Harvard University Press.

[B7] Buszard T. Farrow D. Verswijveren S. J. Reid M. Williams J. Polman R. (2017). Working memory capacity limits motor learning when implementing multiple instructions. *Front. Psychol.* 8:1350. 10.3389/fpsyg.2017.01350 28878701 PMC5572292

[B8] Chambers K. L. Vickers J. N. (2006). Effects of bandwidth feedback and questioning on the performance of competitive swimmers. *Sport Psychol.* 20 184–197. 10.1123/tsp.20.2.184

[B9] Cohen L. Manion L. Morrison K. (2011). *Research Methods in Education.* London: Routledge.

[B10] Côté J. Salmela J. H. Baria A. Russell S. J. (1993). Organizing and interpreting unstructured qualitative data. *Sport Psychol.* 7 127–137. 10.1123/tsp.7.2.127

[B11] Côté J. Salmela J. H. Russell S. (1995). The knowledge of high-performance gymnastic coaches: methodological framework. *Sport Psychol.* 9 65–75. 10.1123/tsp.9.1.65

[B12] Creswell J. W. (2013). *Research Design: Qualitative, Quantitative, and Mixed Methods Approaches.* Thousand Oaks, CA: Sage Publications.

[B13] Davids K. Renshaw I. Pinder R. Greenwood D. Barris S. (2017). “The role of psychology in enhancing skill acquisition and expertise in high performance programmes,” in *Sport and exercise Psychology: Practitioner Case Studies*, eds Weston N. Breslin G. Cotterill S. (Hoboken, NJ: Wiley-Blackwell), 329–353.

[B14] Deakin H. Wakefield K. (2014). Skype interviewing: Reflections of two PhD researchers. *Qual. Res.* 14 603–616. 10.1177/1468794113488126

[B15] Demers G. Woodburn A. J. Savard C. (2006). The development of an undergraduate competency-based coach education program. *Sport Psychol.* 20 162–173. 10.1123/tsp.20.2.162

[B16] Dohrenwend A. (2002). Serving up the feedback sandwich. *Fam. Pract. Manag.* 9 43–46. 12469676

[B17] Dorsch K. D. Etheredge M. (2017). Softball coaches’ perceptions of athlete entitlement. *J. Exerc. Mov. Sport* 49:77.

[B18] Dweck C. S. (2000). *Self-Theories: Their Role in Motivation, Personality and Development.* Hove: Psychology Press.

[B19] Erickson K. Bruner M. W. MacDonald D. J. Côté J. (2008). Gaining insight into actual and preferred sources of coaching knowledge. *Int. J. Sports Sci. Coach.* 3 527–538. 10.1260/174795408787186468 29106335

[B20] Fang Z. (1996). A review of research on teacher beliefs and practices. *Educ. Res.* 38 47–65. 10.1080/0013188960380104

[B21] Gilbert W. D. (2016). *Busting a Culture of Athlete Entitlement. School Sport Canada.* Avaliable at: http://www.schoolsport.ca/busting-a-culture-of-athlete-entitlemen/ (accessed February 26, 2020).

[B22] Greenwood D. Davids K. Renshaw I. (2014). Experiential knowledge of expert coaches can help identify informational constraints on performance of dynamic interceptive actions. *J. Sports Sci.* 32 328–335. 10.1080/02640414.2013.824599 24016400

[B23] Halperin I. Chapman D. W. Martin D. T. Abbiss C. Wulf G. (2016). Coaching cues in amateur boxing: an analysis of ringside feedback provided between rounds of competition. *Psychol. Sport Exerc.* 25 44–50. 10.1016/j.psychsport.2016.04.003

[B24] Harden M. Bruce C. Wolf A. Hicks K. M. Howatson G. (2019). Exploring the practical knowledge of eccentric resistance training in high-performance strength and conditioning practitioners. *Int. J. Sports Sci. Coach.* 15 41–52. 10.1177/1747954119891154

[B25] Hattie J. Timperley H. (2007). The power of feedback. *Rev. Edu. Res.* 77 81–112. 10.3102/003465430298487

[B26] Hattie J. A. C. (2009). *Visible Learning.* London: Routledge.

[B27] Hattie J. A. C. Clarke S. (2018). *Visible Learning: Feedback.* London: Routledge.

[B28] Kahan D. (1999). Coaching behavior: a review of the systematic observation research literature. *App. Res. Coach. Athlet. Annu.* 14 17–58.

[B29] Kluger A. N. DeNisi A. (1996). The effects of feedback interventions on performance: a historical review, a meta-analysis, and a preliminary feedback intervention theory. *Psychol. Bull.* 119 254–284. 10.1037/0033-2909.119.2.254

[B30] Langley D. J. (1997). Exploring student skill learning: a case for investigating subjective experience. *Quest* 49 142–160. 10.1080/00336297.1997.10484230

[B31] Leslie-Toogood A. Martin G. L. (2003). Do coaches know the mental skills of their athletes? Assessments from volleyball and track. *J. Sport Behav.* 26 56–58.

[B32] Liberman A. Liberman M. Steinert Y. McLeod P. Meterissian S. (2005). Surgery residents and attending surgeons have different perceptions of feedback. *Med. Teach.* 27 470–472. 10.1080/0142590500129183 16147804

[B33] Light R. L. Harvey S. (2017). Positive pedagogy for sport coaching. *Sport Educ. Soc.* 22 271–287. 10.1080/13573322.2015.1015977

[B34] Lyle J. (2002). *Sports Coaching Concepts: A Framework for Coaches’ Behaviour.* London: Routledge.

[B35] Mason R. J. Farrow D. Hattie J. A. C. (2020a). An analysis of in-game feedback provided by coaches in an Australian Football League competition. *Phys. Educ. Sport Pedagogy* 2020 1–14. 10.1080/17408989.2020.1734555

[B36] Mason R. J. Farrow D. Hattie J. A. C. (2020b). An exploratory investigation into the reception of verbal and video feedback provided to players in an Australian Football League club. *Int. J. Sports Sci. Coach.* 10.1177/1747954120951080 [Epub ahead of print].

[B37] McQuade S. Davis L. Nash C. (2015). Positioning mentoring as a coach development tool: recommendations for future practice and research. *Quest* 67 317–329. 10.1080/00336297.2015.1048810

[B38] Millar S. K. Oldham A. R. Donovan M. (2011). Coaches’ self-awareness of timing, nature and intent of verbal instructions to athletes. *Int. J. Sports Sci. Coach.* 6 503–513. 10.1260/1747-9541.6.4.503 29106335

[B39] Morris K. S. Jenkins D. G. Osborne M. A. Rynne S. B. Shephard M. E. Skinner T. L. (2019). The role of the upper and lower limbs in front crawl swimming: the thoughts and practices of expert high-performance swimming coaches. *Int. J. Sports Sci. Coach.* 14 629–638. 10.1177/1747954119866358

[B40] Narciss S. Huth K. (2004). “How to design informative tutoring feedback for multimedia learning,” in *Instructional Design for Multimedia Learning*, eds Niegemann H. M. Leutner D. Brunken R. (Münster: Waxmann), 181–195.

[B41] Nash C. Collins D. (2006). Tacit knowledge in expert coaching: science or art? *Quest* 58 464–476. 10.1080/00336297.2006.10491894

[B42] Nicol D. J. MacFarlane-Dick D. (2006). Formative assessment and self-regulated learning: a model and seven principles of good feedback practice. *Stud. Higher Educ.* 31 199–218. 10.1080/03075070600572090

[B43] North J. (2010). Using ‘coach developers’ to facilitate coach learning and development: qualitative evidence from the UK. *Int. J. Sports Sci. Coach.* 5 239–256. 10.1260/1747-9541.5.2.239 29106335

[B44] OECD (2009). *Teaching Practices, Teachers’ Beliefs and Attitudes. In Creating Effective Teaching and Learning Environments.* Paris: OECD, 87–135.

[B45] Parkes J. Abercrombie S. McCarty T. (2013). Feedback sandwiches affect perceptions but not performance. *Adv. Health Sci. Educ.* 18 397–407. 10.1007/s10459-012-9377-9 22581568

[B46] Partington M. Cushion C. (2013). An investigation of the practice activities and coaching behaviors of professional top-level youth soccer coaches. *Scand. J. Med. Sci. Sports* 23 374–382. 10.1111/j.1600-0838.2011.01383.x 22092356

[B47] Partington M. Cushion C. J. Cope E. Harvey S. (2015). The impact of video feedback on professional youth football coaches’ reflection and practice behaviour: a longitudinal investigation of behaviour change. *Reflect. Pract.* 16 700–716. 10.1080/14623943.2015.1071707

[B48] Pashler H. McDaniel M. Rohrer D. Bjork R. (2009). Learning styles: concepts and evidence. *Psychol. Sci. Public Inter.* 9 105–119. 10.1111/j.1539-6053.2009.01038.x 26162104

[B49] Pereira F. Mesquita I. Graça A. (2009). Accountability systems and instructional approaches in youth volleyball training. *J. Sports Sci. Med.* 8 366–373. 24149999 PMC3763281

[B50] Potrac P. Brewer C. Jones R. Armour K. Hoff J. (2000). Toward an holistic understanding of the coaching process. *Quest* 52 186–199. 10.1080/00336297.2000.10491709

[B51] Potrac P. Jones R. Armour K. (2002). ‘It’s all about getting respect’: the coaching behaviors of an expert English soccer coach. *Sport Educ. Soc.* 7 183–202. 10.1080/1357332022000018869

[B52] Putnam R. T. Borko H. (1997). “Teacher learning: implications of new views of cognition,” in *International Handbook of Teachers and Teaching*, eds Biddle B. J. Good T. L. Goodson I. F. (Berlin: Springer), 1223–1296. 10.1007/978-94-011-4942-6_30

[B53] Randell A. D. Cronin J. B. Keogh J. W. Gill N. D. Pedersen M. C. (2011). Effect of instantaneous performance feedback during 6 weeks of velocity-based resistance training on sport-specific performance tests. *J. Strength Condit. Res.* 25 87–93. 10.1519/jsc.0b013e3181fee634 21157389

[B54] Renshaw I. Araújo D. Button C. Chow J. Y. Davids K. Moy B. (2016). Why the constraints-led approach is not teaching games for understanding: a clarification. *Phys. Educ. Sport Pedagogy* 21 459–480. 10.1080/17408989.2015.1095870

[B55] Roberts A. H. Greenwood D. A. Stanley M. Humberstone C. Iredale F. Raynor A. (2019). Coach knowledge in talent identification: a systematic review and meta-synthesis. *J. Sci. Med. Sport* 22 1163–1172. 10.1016/j.jsams.2019.05.008 31133481

[B56] Rushall B. S. (2003). *Coaching Development and the Second Law of Thermodynamics (or belief-based Versus Evidence-Based Coaching Development).* Avaliable at: http://coachsci.sdsu.edu/csa/thermo/thermo.htm (accessed February 26, 2020).

[B57] Rynne S. B. Mallett C. J. (2012). Understanding the work and learning of high performance coaches. *Phys. Educ. Sport Pedagogy* 17 507–523. 10.1080/17408989.2011.621119

[B58] Shute V. J. (2008). Focus on formative feedback. *Rev. Educ. Res.* 78 153–189. 10.3102/0034654307313795

[B59] Smith M. Cushion C. J. (2006). An investigation of the in-game behaviours of professional, top-level youth soccer coaches. *J. Sports Sci.* 24 355–366. 10.1080/02640410500131944 16492600

[B60] Smoll F. L. Smith R. E. (1980). Techniques for improving self-awareness of youth sports coaches. *J. Phys. Educ. Recreat.* 51 46–52. 10.1080/00971170.1980.10624089

[B61] Stoszkowski J. Collins D. (2016). Sources, topics and use of knowledge by coaches. *J. Sports Sci.* 34 794–802. 10.1080/02640414.2015.1072279 26222481

[B62] Tinning R. (1982). Improving coaches’ instructional effectiveness. *Sports Coach.* 5 37–41.

[B63] Woods C. T. McKeown I. Rothwell M. Araújo D. Robertson S. Davids K. (2020). Sport practitioners as sport ecology designers: how ecological dynamics has progressively changed perceptions of skill “acquisition” in the sporting habitat. *Front. Psychol.* 11:654. 10.3389/fpsyg.2020.00654 32390904 PMC7194200

